# Profile and Predictors of Blood Tumor Mutational Burden in Advanced Hepatocellular Carcinoma

**DOI:** 10.1093/oncolo/oyac189

**Published:** 2022-09-14

**Authors:** Joseph W Franses, Mir Lim, Adam M Burgoyne, Kabir Mody, Jochen Lennerz, Jeremy Chang, Robin Imperial, Stacey N Dybel, Thi M Dinh, Jude Masannat, Caroline M Weipert, David Hsiehchen

**Affiliations:** Massachusetts General Hospital and Harvard Medical School, Division of Hematology-Oncology, Boston, MA, USA; University of Texas Southwestern Medical Center, Division of Hematology-Oncology, Dallas, TX, USA; University of California San Diego Moores Cancer Center, Division of Hematology-Oncology, San Diego, CA, USA; Mayo Clinic, Division of Hematology-Oncology, Jacksonville, FL, USA; Massachusetts General Hospital and Harvard Medical School, Department of Pathology, Boston, MA, USA; University of California San Diego Moores Cancer Center, Division of Hematology-Oncology, San Diego, CA, USA; Mayo Clinic, Division of Hematology-Oncology, Jacksonville, FL, USA; Massachusetts General Hospital and Harvard Medical School, Department of Pathology, Boston, MA, USA; Massachusetts General Hospital and Harvard Medical School, Department of Pathology, Boston, MA, USA; Guardant Health, Redwood City, CA, USA; Guardant Health, Redwood City, CA, USA; University of Texas Southwestern Medical Center, Division of Hematology-Oncology, Dallas, TX, USA

**Keywords:** tumor mutational burden, liquid biopsy, hepatocellular carcinoma

## Abstract

Advanced hepatocellular carcinoma (HCC) is responsive to immune checkpoint inhibitors, but there are currently no known biomarkers to predict treatment benefit. Blood TMB (bTMB) estimation via circulating tumor DNA (ctDNA) profiling can provide a convenient means to estimate HCC TMB. Here we provide the first landscape of bTMB in advanced HCC using a commercially available next-generation sequencing assay, show that it is approximately three times as high as matched tissue TMB, and show that bTMB correlates with NAFLD cirrhosis etiology and the presence of genomic alterations in HTERT and TP53. These results lay the foundation for subsequent studies evaluating bTMB as an immune therapy predictive biomarker in HCC.

## Teaser

Blood tumor mutational burden (TMB) estimation via circulating tumor DNA profiling can provide a convenient means to estimate tumor mutational burden for hepatocellular carcinoma. This article reports on blood tumor mutational burden in advanced HCC using a commercially available next-generation sequencing assay.

Hepatocellular carcinoma (HCC) is the sixth leading cause of cancer death in the US and the fourth worldwide.^1^ Treatment of advanced HCC has been revolutionized with the introduction of immunotherapy regimens targeting the PD-1/PD-L1 axis, yet only a subset of patients respond to such therapies. Potential biomarkers of benefit such as PD-L1 expression and microsatellite instability (MSI) are not associated with treatment outcomes or rarely occur in HCC, respectively, and there are extensive ongoing research efforts attempting to identify predictive biomarkers for immune therapy regimens.^2^ Tissue tumor mutational burden (tTMB),^3^ defined as the number of non-synonymous somatic mutations per megabase of genomic DNA in a tumor specimen, may be a proxy for tumor neoantigens and has been associated with checkpoint inhibitor benefit across multiple cancers, possibly by presenting more “non-self” targets for immune system recognition.^4^ The introduction of circulating tumor DNA (ctDNA) profiling has enabled another method to measure TMB, with a parameter called the blood TMB (bTMB). The relationship between bTMB and tTMB has not yet been evaluated in HCC, and it remains unclear whether clinico-genomic factors may be associated with TMB.^5^ In this work, we define the landscape of bTMB in advanced HCC using a commercially available targeted next-generation sequencing (NGS) assay and show that bTMB and tTMB are significantly correlated with bTMB being approximately 3 times as high as tTMB. We also find significant correlations with features such as non-alcoholic fatty liver disease (NAFLD) and mutations in hTERT and TP53. These findings have significant implications in future prospective studies evaluating the use of bTMB in immunotherapy response prediction.

We assembled a cohort of 136 patients across 4 academic cancer centers with unresectable HCC who underwent ctDNA profiling using the Guardant360 assay between October 2020 and July 2021. bTMB quantification was unable to be determined in 14 (10.2%) of these cases. Retrospective collection of patient data for this combined cohort was approved by each center’s institutional review board. Patient demographic data are shown in Supplementary Table S1, and these reflect the expected male predominance (110/136 = 80.9% male) and advanced age (median age 64.6 years) of disease onset. tTMB scores were determined in 35 (25.7%) patients, of which 28 patients also had successful bTMB testing.

**Figure 1.**
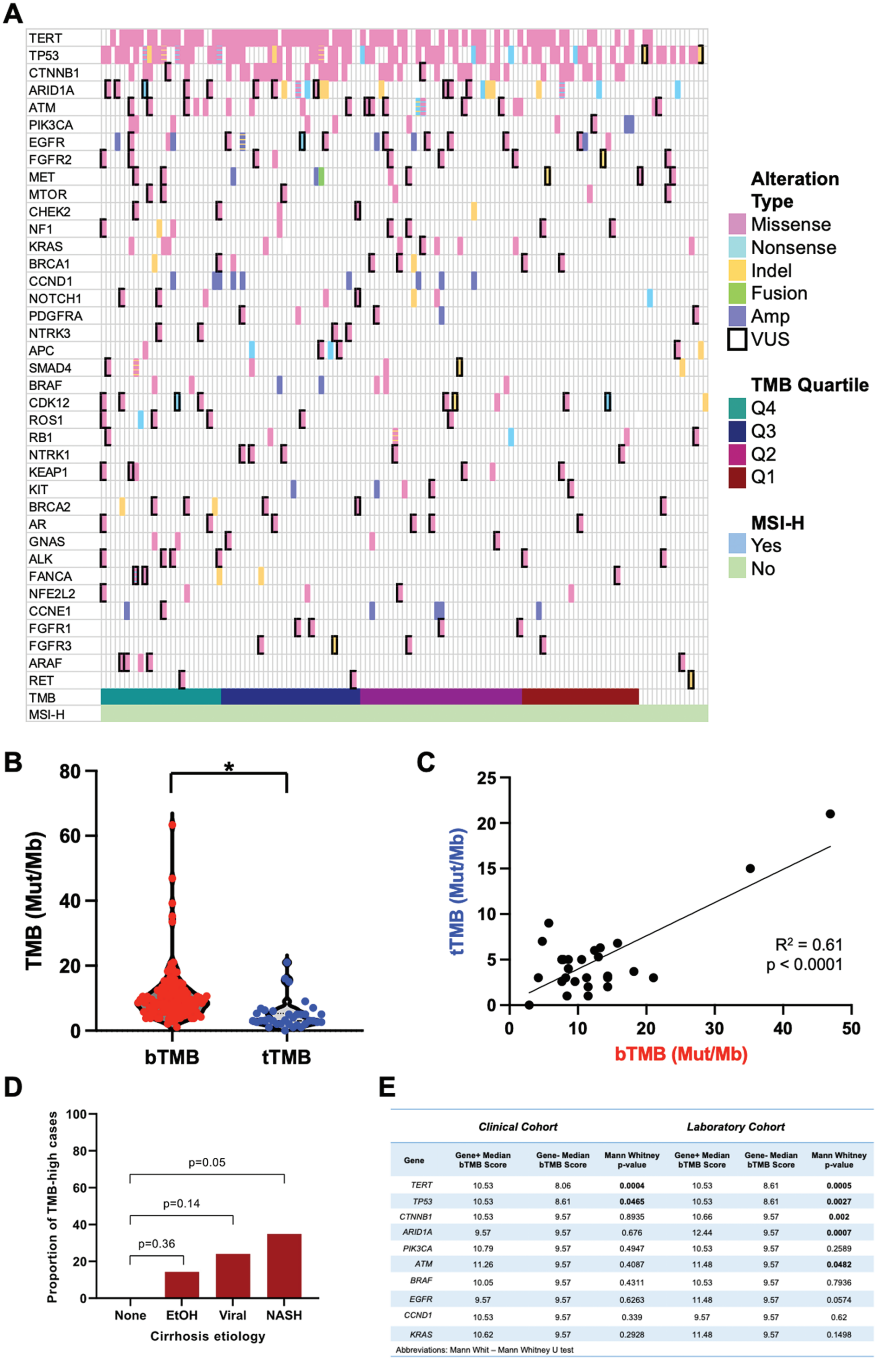
TMB estimates from ctDNA are significantly different from—but correlated with—estimates from tissue. (**A**) Oncoprint diagram of genomic alterations observed in the clinical cohort, listed in descending order of bTMB. (**B**) Violin plot of bTMB and tTMB for 28 of the patients with matched liquid and solid specimens. *P* < .0001 by unpaired *t*-test. (**C**) Scatter plot of tTMB versus bTMB for the matched specimens. (**D**) Compared to patients with no clear cirrhosis etiology, there was a higher proportion of patients with high bTMB in patients with NASH cirrhosis. (**E**) Genomic alterations associated with higher TMB in the clinical cohort and the wider laboratory cohort.

The ctDNA profiles of HCC in this cohort demonstrated the expected distribution of HCC genomic alterations,^6^ with frequent alterations in *TP53*, *CTNNB1*, and *TERT* (Fig. 1A). MSI-high status was identified in zero patients, consistent with the low frequency of this feature in HCC.^3^ The bTMB mean and median were 10.6 Mut/Mb and 8.6 Mut/Mb, respectively, whereas the tTMB mean and median were 4.8 and 3.0 (*P* < .0001, unpaired *t*-test; Fig. 1B). bTMB and tTMB samples were taken a median of 12 days apart (range of tissue sample date minus blood sample date: −1036 to +285 days).^3,7^ For the 28 patients that had matched bTMB and tTMB, and in whom the bTMB and tTMB distributions were similar to that of the larger cohort (Supplementary Fig. S1A), we found a significant correlation (*R*^2^ = 0.61, *P* < .0001; Fig. 1C), with a linear regression slope of 2.7. We confirmed that the distribution of bTMB in our clinical cohort was representative of the broader cohort in the company database (Supplementary Fig. S1B).

To determine whether clinical or genomic factors may be associated with TMB, we assessed the relationship between disease etiology, demographics, and frequent driver gene mutations. bTMB was significantly associated with disease etiology (Kruskal–Wallis test, *P* = .016), with HCC associated with non-alcoholic fatty liver disease (NAFLD) having the highest mean bTMB and proportion of TMB-high cancers (bTMB in the fourth quartile; Fig. 1D). bTMB was also associated with age (Mann–Whitney test, *P* = .002), with patients at least 65 years old having higher median bTMB and proportions of TMB-high cancers (Supplementary Fig. S3C). In contrast, biological sex and race/ethnicity and were not associated with bTMB (Supplementary Fig. S3D, E). In our dataset, genomic alterations in *TERT* and *TP53*—the 2 most common genes altered in HCC—correlated with higher bTMB, and these were confirmed in the broader laboratory dataset (Fig. 1E). In the laboratory dataset, alterations in *CTNNB1, ARID1A*, and *ATM* were also correlated with higher bTMB.

In summary, we present, for the first time, the landscape of bTMB in advanced HCC using a targeted NGS assay and provide a direct comparison of matched bTMB and tTMB in a subset of our cohort. We found that bTMB and tTMB are significantly different, with bTMB approximately 2.7 times as high as tTMB, with this observation being concordant with previous studies in other histologies.^8^ Differences in bTMB versus tTMB may stem from technical factors (differences in the size/location of sequenced genome regions and algorithms for TMB calculation) or from intrinsic biological mechanisms. Given that ctDNA samples genomic DNA from multiple tumor foci,^9^ and various tumor foci may develop distinct mutational profiles as they evolve under different conditions,^10^ ctDNA may provide a higher estimate of TMB when compared with tTMB measured from a specific tissue site. Indeed, for lung cancer, tTMB is significantly heterogeneous both within a single primary tumor and between a primary tumor and metastatic sites.^11^ The bTMB and tTMB scores were also calculated a median of 12 days apart, suggesting that tumor evolution and treatment may contribute to these differences. Nonetheless, our results indicate that bTMB may be a reliable surrogate for tTMB in advanced HCC.

Our study’s strengths included the fact that it is the first of its kind for HCC, that our patients had tTMB measurements that were similar to prior literature estimates^3^ leveraging larger tissue datasets, and that we included data from a heterogeneous patient population from 4 geographically distinct academic cancer centers. The latter point may also illustrate significant study heterogeneity. Additional study weaknesses included the limited sample size and retrospective nature of the study, the incomplete matching of bTMB and tTMB across the entire bTMB dataset, the use of disparate tTMB sequencing platforms at each institution, the differences in collection dates of bTMB and tTMB for some of the patients, and the lack of a direct assessment of immune therapy response utilizing the bTMB measurements that we collected. The last point will be an area of significant future interest for our groups and the broader research community.

## Supplementary Material

oyac189_suppl_Supplementary_Material

## Data Availability

Deidentified patient data from the clinical cohort will be provided to interested parties upon request.
