# Supplementary material for: Profile and Predictors of Blood Tumor Mutational Burden in Advanced Hepatocellular Carcinoma
Source: Oncologist. 2022 Sep 14;27(11):e908–11. doi: 10.1093/oncolo/oyac189 (PMC9632309; doi:10.1093/oncolo/oyac189)
Supplement: oyac189_suppl_Supplementary_Material [file oyac189_suppl_supplementary_material.pdf]

Supplementary material for "Profile and predictors of blood tumor mutational burden in advanced hepatocellular carcinoma"

Supplementary Methods

ctDNA Analysis in the Clinical Cohort

All patients in the clinical cohort received ctDNA analysis as part of usual clinical care via a commercially available, targeted next-generation sequencing assay (Guardant360, Guardant Health). Guardant360 is a CLIA-accredited, College of American Pathologists-approved, New York State Department of Health-approved cfDNA assay with analytic and clinical validation previously reported.[1] During the study period, the assay included clinical reporting of 83 genes plus microsatellite instability, with coverage of single nucleotide variants (SNVs) and select insertions/deletions, amplifications, and fusions over a larger genomic footprint to provide a bTMB score. The algorithm and analytic validation for the bTMB score generated as part of this assay has been previously described.[2]

Data Analysis of Larger HCC Laboratory Cohort

To assess bTMB values and gene frequency association in a larger population of HCC patients, we queried a de-identified database containing results from consecutive patients who underwent clinical testing with Guardant360. Patients with a diagnosis of HCC, as reported by the ordering physician on the test requisition form, tested between October 2020 and March 2022 using the panel version with bTMB score available were included in this analysis. All patients had advanced disease (stage IIIB or higher) as reported by the ordering physician. This research was approved by the Quorum Institutional Review Board (IRB) for the generation of deidentified data sets for research purposes.

Statistical Analyses

Median bTMB scores were calculated within both the clinical cohort and laboratory cohort comparing samples with versus without pathogenic alterations in the most frequently mutated genes. Median bTMB scores were compared using a Mann Whitney U test. These analyses were not mutually exclusive (i.e. samples with co-occurring pathogenic TERT and TP53 alterations could be included in the median bTMB calculation for each gene). Statistical analyses were done using GraphPad Prism version 9.3.1 for macOS, GraphPad Software, San Diego, California, USA, www.graphpad.com.

Supplementary References

1. Zill OA, Banks KC, Fairclough SR, et al. The Landscape of Actionable Genomic Alterations in Cell-Free Circulating Tumor DNA from 21,807 Advanced Cancer Patients. Clin Cancer Res 2018;24:3528-3538.
2. Si H, Kuziora M, Quinn KJ, et al. A Blood-based Assay for Assessment of Tumor Mutational Burden in First-line Metastatic NSCLC Treatment: Results from the MYSTIC Study. Clin Cancer Res 2021;27:1631-1640.

Age	64.0	IQR 59.0 – 72.0
Sex	19.1%	female
	80.9%	male
Ethnicity	10.3%	Asian
	7.4%	Black
	243.%	Hispanic
	51.5%	White
	6.6%	Other
Cirrhosis	18.4%	Alcohol
etiology	33.1%	NAFLD
	37.5%	Viral
	11.0%	Other
Disease stage	2	4.4%
	3	44.1%
	4	50.7%

Table S1. Patient demographics for this study.

A

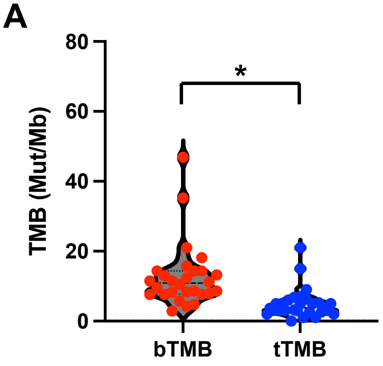

B

Clinical Cohort		Laboratory Cohort	
Minimum	1.13	Minimum	1.91
25th Percentile	6.29	25th Percentile	6.70
Median	9.57	Median	9.57
75th Percentile	13.4	75th Percentile	14.35
Maximum	63.36	Maximum	148.7
Mean	11.46	Mean	11.96
Standard Deviation	8.55	Standard Deviation	10.87

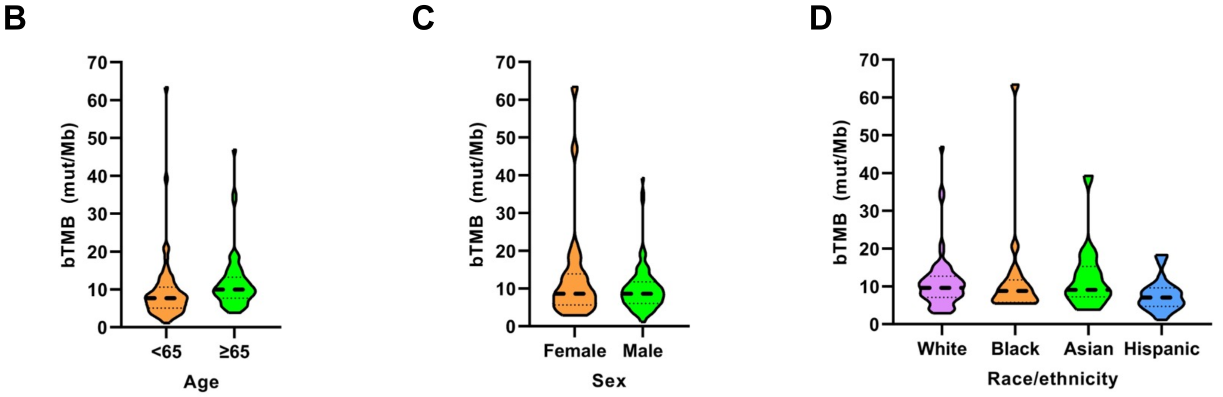

Figure S1. (A) bTMB and tTMB in the patients with matched samples were similar to those of the broader cohort. (**B**) Descriptive statistics of bTMB derived from the clinical multi-institutional cohort and the overall Guardant Health database. (**C, D, E**) bTMB distributions based on participants' age (C), biological sex (D), and self-described race/ethnicity (E).
